# Rare variants in non-coding regulatory regions of the genome that affect gene expression in systemic lupus erythematosus

**DOI:** 10.1038/s41598-019-51864-9

**Published:** 2019-10-28

**Authors:** Sarah A. Jones, Stuart Cantsilieris, Huapeng Fan, Qiang Cheng, Brendan E. Russ, Elena J. Tucker, James Harris, Ina Rudloff, Marcel Nold, Melissa Northcott, Wendy Dankers, Andrew E. J. Toh, Stefan J. White, Eric F. Morand

**Affiliations:** 1 Centre for Inflammatory Diseases, Department of Medicine, School of Clinical Sciences, Monash University, Clayton, Victoria 3168 Australia; 2 Department of Molecular and Translational Science, Monash University, Clayton, Victoria 3168 Australia; 3 Department of Microbiology, Biomedical Discovery Institute, Monash University, Clayton, Victoria 3800 Australia; 4 Murdoch Children’s Research Institute, Royal Children’s Hospital, Parkville, Victoria 3052 Australia; 5 Department of Paediatrics, University of Melbourne, Parkville, Victoria 3052 Australia; 6 Hudson Institute of Medical Research, Clayton, Victoria 3168 Australia

**Keywords:** Genetics research, Systemic lupus erythematosus

## Abstract

Personalized medicine approaches are increasingly sought for diseases with a heritable component. Systemic lupus erythematosus (SLE) is the prototypic autoimmune disease resulting from loss of immunologic tolerance, but the genetic basis of SLE remains incompletely understood. Genome wide association studies (GWAS) identify regions associated with disease, based on common single nucleotide polymorphisms (SNPs) within them, but these SNPs may simply be markers in linkage disequilibrium with other, causative mutations. Here we use an hierarchical screening approach for prediction and testing of true functional variants within regions identified in GWAS; this involved bioinformatic identification of putative regulatory elements within close proximity to SLE SNPs, screening those regions for potentially causative mutations by high resolution melt analysis, and functional validation using reporter assays. Using this approach, we screened 15 SLE associated loci in 143 SLE patients, identifying 7 new variants including 5 SNPs and 2 insertions. Reporter assays revealed that the 5 SNPs were functional, altering enhancer activity. One novel variant was linked to the relatively well characterized rs9888739 SNP at the ITGAM locus, and may explain some of the SLE heritability at this site. Our study demonstrates that non-coding regulatory elements can contain private sequence variants affecting gene expression, which may explain part of the heritability of SLE.

## Introduction

Systemic lupus erythematosus (SLE, or lupus) is the archetypal multisystem autoimmune disease. SLE patients are predominantly young women who suffer a marked loss of life expectancy and severe morbidity^1^. The causes of SLE are heterogeneous and poorly defined, and patients are routinely treated with broad-spectrum immunosuppressive therapies associated with a high risk of infection, cardiovascular disease, osteoporosis, bone marrow suppression and infertility. Due to the heterogeneity of the disease and the limited knowledge of causative factors, a number of high profile clinical trials of targeted SLE therapies have yielded negative results^2^. Better identification of the causative factors of SLE would allow the development of acutely needed biomarkers, targeted therapies, and potentially personalized medicine approaches^3^.

Considerable evidence supports a genetic contribution to the development of SLE. Twin studies indicate 25–40% concordance for SLE in monozygotic twins, versus 2% concordance in dizygotic twins^4^. Significant effects of ethnicity on SLE disease severity have also been reported, for example both Indigenous Australians and patients of Asian ethnicity have markedly increased SLE prevalence and severity^5^. Microarrays and high-density single nucleotide polymorphism (SNP) genotyping allow genome-wide association studies (GWAS) to be performed on thousands of SLE DNA samples and such studies have implicated several dozen loci in SLE susceptibility^6,7^. Currently, GWAS studies are estimated to explain 50% of the heritability in SLE^8^. Although the effect of some disease-associated SNPs can be explained by effects on the coding sequence of a gene, >80% of SLE-associated SNPs are in non-coding DNA^6^. Interestingly, the non-coding regions containing SLE-associated SNPs show an enrichment in enhancer-associated histone modifications, suggesting their potential importance in driving gene expression and SLE pathogenesis^8^. Indeed several non-coding variants have been functionally validated using techniques such as luciferase reporter assays and transcription factor binding analysis^9–13^. The studied SNPs modulate transcription factor binding strength and can thereby affect gene transcription of nearby genes, but also of genes further away via long-range chromatin interactions^10^. However, these studies only covered a small proportion of all the SLE-associated SNPs in non-coding regions.

Genome-wide association studies can identify SNPs in non-coding regions, but once such a variant is identified, two important factors need to be considered. Firstly, is the SNP causative or just a marker in linkage disequilibrium with the true functional mutation? This can be answered by searching for secondary SNPs in linkage disequilibrium with the associated SNP^14^ or by bioinformatic screening of GWAS-associated regions to identify putative regulatory elements, which are then more finely combed for variants^15^.

A second issue arises once a variant is identified in non-coding DNA. How can the variant be screened in a bioinformatics approach for potential functionality? Attributing a functional effect to non-coding DNA variants is more challenging than for variants in coding DNA. However, predictive tools can be used to map loci that are potential regulatory sites, allowing the identification of non-coding regions that are likely to impact on gene expression. For example, the RegulomeDB database^14^ integrates factors such as histone modifications, open chromatin, predicted transcription factor binding sites (TFBS) and measured transcription factor binding to estimate the likelihood that a particular genetic variant will affect binding of proteins to the DNA.

Here, we demonstrate an approach that addresses these two factors. We hypothesized that by identifying regions that are predicted sites of transcriptional regulation based on their RegulomeDB score, which are located within loci identified previously by SLE GWAS analyses, we could narrow down the area to be searched for causative mutations, thus allowing identification of novel, functional, variants implicated in SLE susceptibility. We identified 5 such variants, and moreover, showed these variants to have functional effects on gene expression that may be predicted to influence SLE pathogenesis. One such variant was found near the rs9888739 SNP in the ITGAM locus and, like rs9888739, inhibited ITGAM expression. The contribution of dysregulated ITGAM expression in SLE may be, at least in part, due to the novel SNP we identified here.

## Results

We hypothesised that in some cases, GWAS studies may identify SNPs that act as markers of susceptibility loci, but which are not in fact the functional polymorphism. In such cases, other unidentified variants that contribute to disease risk may lie in close proximity to the identified SNP, and are essentially masked from discovery and characterisation by their localisation proximal to the existing annotated SNP. Such ‘hidden’ variants have been proposed to contribute to the missing heritability in SLE^16^. To identify novel rare variants in patients with SLE, we first chose regions identified as SLE susceptibility loci in GWAS studies, then selected loci based on their being predicted regulatory regions as indicated in the RegulomeDB database. These candidate loci containing the GWAS-identified SLE risk SNPs were screened for nucleotide polymorphisms in addition to the previously identified SNP using high resolution melt (HRM) analysis. DNA samples from 143 patients with SLE were screened by HRM for variants in 15 loci previously linked with SLE. As all of these loci contained common SNPs, multiple HRM curves were generated for each sample. In most cases there were three major curves per locus, corresponding to homozygous reference sequence, homozygous variant, and heterozygous reference/variant. As the focus of the research was the identification of rare variants, only curves present in 1–2 DNA samples were chosen for further analysis. Sanger sequencing of each candidate locus revealed seven rare variants (defined as either previously undescribed, or only found in a single individual), five single nucleotide variants and two insertions (Table 3).

To determine effects of the variants on gene expression, luciferase assays were performed by transfecting constructs containing each of the rare variants into a cell line, and measuring the amount of luciferase produced. Importantly, cloned sequences did not contain the SNP used to identify the region of interest originally, and thus we are able rule out the possibility of that SNP being responsible for any changes in reporter gene expression. All reference sequences were associated with significantly increased luciferase activation relative to a control transfection, consistent with the cloned DNA fragment having regulatory activity in the cell type. All of the rare variants gave a RegulomeDB score at least as likely to impact binding as the corresponding SNP.

### A novel variant at the ITGAM locus

**Figure 1:**
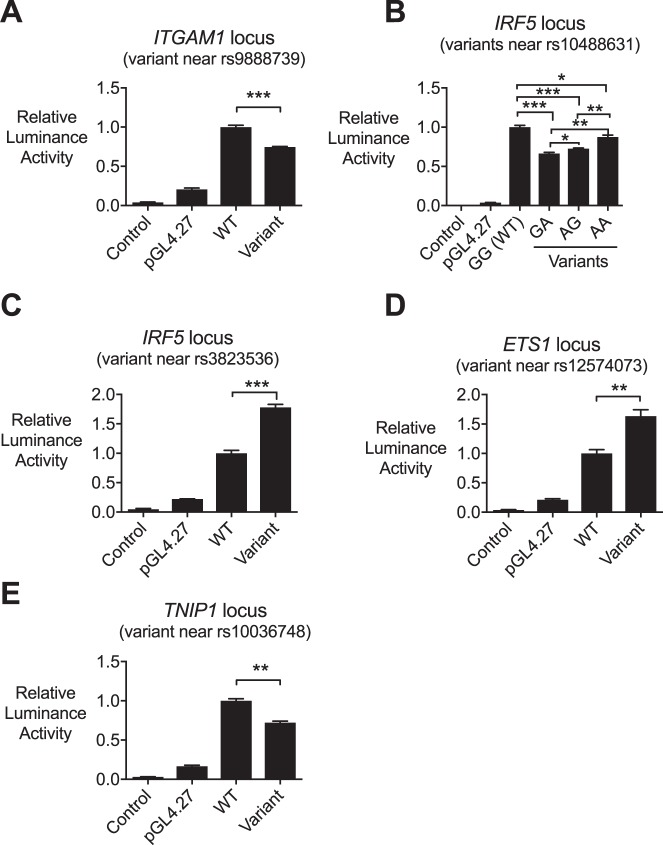
Luciferase assays showing effects of novel variants on gene expression. Novel variants were cloned into luciferase reporter constructs and assayed for their effects on luciferase activity as an indicator of their effects on expression of their linked gene. Control = no transfection. Assays were repeated four times and representative results are shown. *P < 0.05, **P < 0.01, ***P < 0.001.

Using our targeted sequencing approach, a rare variant was identified in the ITGAM locus, in linkage disequilibrium with rs9888739, found in GWAS studies to associate with SLE susceptibility^17,18^. The novel variant inhibited ITGAM expression (Fig. 1A; raw data from luciferase assays shown in Supplementary Table 1), matching the reported impairment of ITGAM expression in association with SLE-associated alleles. ITGAM encodes the CD11b chain of the Mac-1 integrin complex (alphaMbeta2; CD11b/CD18; complement receptor-3) and in the context of SLE, ITGAM expression may be protective through mediation of phagocytosis of iC3b-opsonised apoptotic material, inhibition of T cell activation, restriction of toll-like receptor signaling and inhibition of Th17 responses^19^.

**Figure 2:**
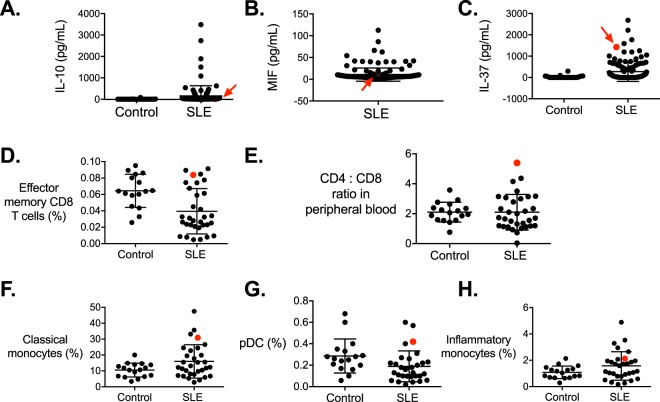
Phenotypic characterization of patient bearing novel variant in the *ITGAM1* locus. Sera from healthy control donors and patients with SLE, including the individual bearing the novel mutation in the *ITGAM1* locus (highlighted in red and indicated with red arrows) was assayed for IL-10 (**A**), MIF (**B**) and IL-37 (**C**) No healthy control donor data was available for MIF levels but this data has previously been published^26^. (**D**) Effector memory CD8 T cells in peripheral blood of healthy control donors and patients with SLE, and (**E**) the ratio of total CD4 to CD8 T cells in PBMC. Proportions of classical monocytes (**F**), plasmacytoid dendritic cells (pDC, **G**) and inflammatory monocytes (**H**) in PBMC. Bars show mean +/−standard deviation. For (**A**–**C**), n = 114, 159 and 127 respectively. For (**E**–**H**), n = 32 SLE patients and 16 HC.

Previous studies of ITGAM variant rs1143679 had found this allele to be associated with increased risk of renal disease, discoid rash, and immunological manifestations^20,21^. The patient bearing the novel ITGAM variant we identified had a history of proteinuria and pyuria, arthritis but no discoid rash, based on a 5.5 year period of follow up. The patient was B lymphopenic, had anti-dsDNA antibodies and low complement. Further examination of their immunological profile showed some abnormalities when compared with a larger cohort of SLE patients we have described elsewhere^22^. The patient had no significant difference in levels of circulating interleukin 10 (IL-10) or macrophage migration inhibitory factor (MIF; Fig. 2A,B), clinical associations of which we have previously described^23–26^. However, the patient bearing the novel C > G variant had substantially higher levels of IL-37 in serum than other SLE patients (1421 pg/mL compared with mean +/−SD of 277 +/−464 pg/mL in a group of 127 SLE patients studied, described previously)^23^, (Fig. 2C). IL-37 is an anti-inflammatory cytokine strongly up-regulated in monocytes by TLR ligation and positively correlated with SLE disease activity^23,27^.

When examining cell populations in the circulation of the patient bearing the novel variant in the ITGAM locus, some differences from the SLE cohort (described in)^22^ were observed. While naïve CD8 and CD4 T cell frequencies were unaffected, effector memory CD8 T cells were elevated at 0.0837% of PBMC, outside the upper 95% CI (0.0752%) of the mean (0.0536%) of the SLE cohort (Fig. 2D), and the ratio of total CD4:CD8 T cells in peripheral blood of the patient bearing the variant was substantially higher than all other SLE patients studied (n = 32) (Fig. 2E). The patient also had a greater proportion of classical monocytes (30.9% compared with mean +/−SD of 10.54 +/−4.37% of the cohort), and plasmacytoid dendritic cells (0.42% compared with mean +/−SD of 0.286 +/−0.160), but no difference in inflammatory monocyte proportions (Fig. 2F).

### Novel variants in IRF5 locus

**Figure 3:**
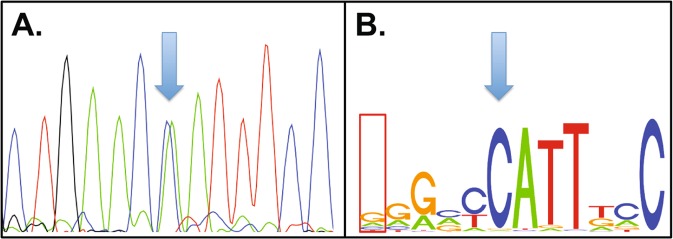
Novel variant in the region of rs10488631, 3′ of *IRF5*. (**A**) The variant identified in DNA from an SLE patient. The arrow indicates the heterozygous variant. (**B**) The TFBS consensus motif containing rs10488631 and the new sequence variant. The box at the left of the motif indicates the position of rs10488631, and the arrow indicates the position of the new variant identified in our study.

Confirming our approach, we identified another novel rare variant in the same predicted TFBS as rs10488631, which is located 3′ of IRF5. At this locus, a G to A substitution was identified in one patient, and another G to A mutation, 89 nucleotides downstream, was identified in a separate patient. Luciferase assays showed both G to A variants to decrease IRF5 gene expression but this effect was not additive if both variants were present (Fig. 1B and Supplementary Table 1). A role for rs10488631 in SLE has been suggested by several studies, and it has also been implicated in other autoimmune conditions such as systemic sclerosis, Sjogren syndrome and rheumatoid arthritis^28–33^. The first of these rare variants was in a high information nucleotide within the consensus sequence for NANOG (Fig. 3A), in contrast to rs10488631 where the affected nucleotide is less invariant (Fig. 3B). NANOG is a TF involved in stem cells, and plays a role in regulating pluripotency. There was a second putative TFBS listed in this locus, which is predicted to bind EHF. EHF is part of the ETS TF family, several members of which have previously been implicated in SLE. EHF plays a role in dendritic cell differentiation, and a GWAS has previously associated EHF with SLE in Europeans^34^.

Three other variants were within a predicted TFBS close to, but separate from, the TFBS containing the common SNP. One was located 5′ of the *IRF5* gene, near rs3823536 (in linkage disequilibrium with rs4728142, which was previously linked to SLE^35^). The rare variant disrupts a high information nucleotide in a CACD motif, which can also bind the transcription factor SP1^36^, and luciferase assays showed the novel variant to increase IRF5 expression (Fig. 1C). SP1 is particularly interesting in the context of IRF5 and SLE, as a previous study of the upstream region of IRF5 identified a 5 bp indel polymorphism, creating an additional SP1 binding site, that was associated with SLE^37^. SP1 binding at other loci has also been implicated in SLE^38^.

### Variant at the ETS1 locus

We identified an additional novel variant located near rs12574073, which is in linkage disequilibrium with rs1128334. These variants are at the 3′ end of *ETS1* (as with EHF a member of the ETS family). ETS1 is involved in B cell and Th17 cell differentiation, and an association between rs1128334 and SLE has been reported in Asian SLE cohorts^39^. Another SNP downstream of *ETS1*, rs6590330, was also implicated in SLE in an independent study^35^. The rare variant we identified at this locus was located in a lower information nucleotide in a FOXP3 motif and was found to increase ETS1 expression via luciferase assay (Fig. 1D and Supplementary Table 1). T regulatory cells are characterized by FOXP3 expression, and inhibition of FOXP3 leads to induction of the Th17 pathway, which is known to contribute to SLE pathogenesis^25^. While regulation of FOXP3 by ETS1 is established^40^, a reciprocal regulatory relationship is not. However, ETS1 and FOXP3 mRNA levels were both reduced and positively correlated with each other in Treg cells from SLE patients^41^.

The patient bearing the novel variant in the ETS1 locus was diagnosed at age 12. Over an 8-year observation period, the patient experienced arthritis, haematuria and lymphopenia, but had not (yet) displayed anti-cardiolipin antibodies, discoid lesions, vasculitis or thrombocytopenia, disease manifestations that had previously been associated with various ETS1 alleles in SLE patients^42^.

### Variant at the TNIP1 locus

We found a rare variant near rs10036748, within the *TNIP1* gene, which impaired TNIP1 expression according to a luciferase reporter assay (Fig. 1E and Supplementary Table 1). An association for rs10036748 with SLE was made in a Chinese cohort, and TNIP1 is thought to play a role in SLE through the NFKB pathway^43^. The rare variant was located in a Gfi1/Gfi1b motif. The affected nucleotide was low information in the Gfi1 sequence, but high information in the Gfi1b motif. TNIP1 is a negative regulator of NFκB signaling and polymorphisms in its locus have been associated with a large number of autoimmune diseases. A mouse strain bearing mutant TNIP1, unable to bind ubiquitin, developed a lupus-like phenotype^44^. Moreover, mice lacking Gfi1 have recently been reported to develop a TLR7-dependent lupus-like phenotype, which the authors showed to involve excess NFκB signaling^45^.

## Discussion

Many studies have shown that a significant proportion of common variants associated with disease are located in genomic regions thought to play a role in regulating gene expression^46^. Less well studied, however, is the impact of rare variants on gene regulation. In this study we identified seven rare variants, including five SNPs and two indels. Four of the five rare SNPs identified in this study were within predicted TFBS. A previous report of common, non-coding variants in autoimmune conditions such as SLE found that most of the candidate variants were positioned outside the TFBS^47^. It has been shown in the hemoglobin locus that sequence variants outside TFBS are still capable of disrupting TF binding, presumably through an effect on local chromatin structure^48^. Additionally, high-resolution analyses of TF binding using ChIP-Nexus has shown that the TF footprint extends further than the binding motif, further evidence that sequence variants outside the TFBS can impact on TF binding^49^. It is plausible that most variants within the TFBS will have a stronger effect on binding, and may therefore be under negative selection.

This study targeted loci previously implicated in disease susceptibility through GWAS analysis of common variants. We screened 16 small (~200 bp) genomic regions, and identified a number of rare variants that were in each case at least as likely to have an impact on regulatory potential as the previously associated, common variants as predicted by RegulomeDB. We identified novel variants in the loci of IRF5, ETS1, ITGAM1 and TNIP1, each of which caused alterations in the expression levels of the association genes. Alone, these novel variants are unlikely to be a major contributor to SLE susceptibility at a population level. However, combined with the multitude of other (common and rare) variants in relevant regulatory regions, demonstration that they are functional suggests they will likely play a role in the disease. Identifying which of the millions of variants in every human genome are involved in disease expression is a major challenge in human disease genetics.

Although complete genome sequencing is becoming more affordable, sequencing thousands of samples is still out of the reach of most laboratories, and the vast majority of variants will not play a role in a given disease. Analysing cell-specific epigenetic data allows prioritization of non-coding sequences that may have a role in a specific disease. A study using H3K27Ac (a known marker of active enhancers) enriched loci across a range of cell types found that the most important cell types for SLE appear to be T cells and particularly B cells^47^. The importance of B cells is further underlined by a report that integrated gene expression data of different immune cell types with GWAS data of autoimmune diseases, and found that all significant associations were with B cell subsets^50^. By selecting all putative regulatory elements in relevant cells and focusing variant screening solely on these regions, it will be possible to identify the majority of the genetic variants that potentially play a role in SLE. Our findings indicate that for a subset of patients, potentially disease-associated functional rare variants can be identified using a targeted sequencing approach focusing on regulatory regions associated with previously identified common variants.

## Methods

### Ethical approval and informed consent

For experiments involving human samples, all samples were collected using protocols approved by the Monash Health Ethics Committee. All experiments were performed in accordance with relevant guidelines and regulations. Informed consent was obtained from all participants.

### Selection of loci

A selection of SNPs identified as SLE susceptibility loci in GWAS studies (referred to in Table 2) were chosen for investigation for novel rare variants based on their being in putative regulatory regions as indicated in the RegulomeDB^14^ website (http://regulomedb.org/GWAS/index.html). The selected loci were screened by high resolution melt analysis (below) for additional unidentified SNPs (not overlapping, but in linkage disequilibrium, with the GWAS SNP).

### High resolution melt analysis

Primers for amplification of each locus, corresponding with Human Genome hg19 notation, are listed in Table 1. High resolution melt (HRM) analysis was performed as described previously^51^. In brief, PCR amplifications were performed in 10 µL reaction volumes, consisting of HRM Master Mix (Idaho Technologies, USA), 5 µM each of forward and reverse primer, and 25 ng genomic DNA. PCR products were analysed in a 96 well plate in the LightScanner (Idaho Technologies, USA). The HRM settings for the LightScanner were as follows; start temperature of 70 °C, end temperature at 96 °C, with a hold temperature at 67 °C. HRM curves were normalized using GeneMelt software supplied with the instrument. Aberrant curves were identified by visual analysis and selected PCR products underwent Sanger Sequencing.

**Table 1:** Primers for amplification of each locus.

Common SNP ID	Sequence Forward	Sequence Reverse	Gene	Location GRCh37	Regulome DB Score
rs13277113	GAGCTTCAGGCAAGATGTCC	CCAGTCCAAGATTCACCTCAG	*BLK*	chr8:11349106-11349337	5
rs2618476	CACTCGGCCTCTTGATAGGA	CAGTTGGTGTTTCCTGGTGA	*BLK*	chr8:11352441-11352669	1d
rs2736335	GTGCAATCAGTGTTGGCTGT	TTGGTTGGTGTTTTTGTCCA	*BLK*	chr8:11341434-11341669	4
rs969985	CAGCAGCCAGAGCTTACTGA	ACAGCCAACACTGATTGCAC	*BLK*	chr8:11341211-11341453	2b
rs12574073	GGCCCTGTTGTGTGATACCT	ATGGCCTGTTCTTGGCTCTA	*ETS-1*	chr11:128319404-128319593	3a
rs11185603	GCTCAACTGGAACTGGGAAG	GAGCTCGTTGTTGTGTGGTG	*IKZF1*	chr7:50306738-50306937	2b
rs3823536	TGTACAGGGAACCCCTTGTC	CTGGAGTCCCAGGAGACAGT	*IRF5*	chr7:128579542-128579750	2b
rs752637	GAAACTGTAGCCCCTCAGGA	CAAAAGGTGCCCAGAAAGAA	*IRF5*	chr7:128579213-128579449	1b
rs10488631	CAGGTACCAAAGGCTGCTTC	TGAGGGCACTGTTCTGTCTG	*IRF5/TNPO3*	chr7:128594148-128594325	1f
rs9888739	CACCCATATCATGGCTTCAGA	GAAAGAACCATGAGCATGAGC	*ITGAM*	chr16:31313154-31313407	1f
rs9888879	GGTTCCATCTTCCCTGTTCA	GCTGTACAACATTGCACCAA	*ITGAM*	chr16:31310286-31310508	2b
rs3130320	GGCTGAGTCACAGGGAAGAA	ACACAGAGACCCACGAGCTT	*NOTCH4*	chr6:32223144-32223381	3a
rs34202539	CAGCATGGTGTGACCAAATC	GGATACCCCCACCAGTTTTT	*TNIP1*	chr5:150458354-150458578	4
rs1150754	ACTGTCACACCCCTCCTCAC	GCGGTTGGACTTGTCAGATT	*TNXB*	chr6:32050679-32050949	4
rs140489	GGCAAGTCACTGGCTTCTTC	CAAGGAAGCCAAATTGAGGA	*UBE2L3*	chr22:21921209-21921364	5

**Table 2:** Sequences that were cloned for functional validation.

Common SNP ID	Ref.	DNA sequence with variant site (underlined and capitalized)	Gene Implicated	Variant 1 (Var1)		Variant 2 (Var2)	
rs9888739	^17^	GGTTCCATCTTCCCTGTTCAtattctttc**C**caccatagccacctgagaccatctagttttctggcctctggtctctgggtttttgctagccttacatttttctttctttatgtttaaaaatttttttattgtggtaagggcacttaacatgagacctatcctcttaacagattttaaaatgtacaatgtaatactgtcatctaTTGGTGCAATGTTGTACAGC	ITGAM	C		G	
rs10488631	^52^	TGTACAGGGAACCCCTTGTCctctccctgagctgg**G**tgtgggtttgcaaggagacatgtgacccagaccaaccctgggagcagcagggcgcctgctgtctggccactcttactaggactgctgt**G**gcacttcctcccctagtgggtccctggtgcccatgaattgcagctcctgggtggtggtgggggcACTGTCTCCTG GGACTCCAG	IRF5	Var1	Var2	Var3	Var4
				GG	GA	AG	AA
rs3823536	^35^	CAGGTACCAAAGGCTGCTTCcatagctagtctagctgaacCatttccgagctacaaggcagtgaatgaaagtaaaaacaaagaaacactggttaaattttaaaaatttattctttctcttttgttgctgttgatttgttcttgagatggctacaacacCAGACAGAACAGTGCCCTCA	IRF5	C		A	
rs12574073	^39^	GGCCCTGTTGTGTGATACCTtctgacacatacgtttttttgaaaaaagattgtctgctgggaactggactgaaaccaacatataac**C**gtttgtttcatactggttaggaagccaccaggaaggcctacccaaagtggttttaaatacatacacacacagtcctctcctctTAGAGCCAAGAACAGGCCAT	ETS1	C		T	
rs10036748	^35^	CAGCATGGTGTGACCAAATCacag**C**gggtacaggagtaaaacagtaaccagtgggttggagagagaggcagacaaacaacctcctacaacgctgcctctctcaaatcaggtcggcctgacccaaccaggacatccgggccccaagtcacaggcagcactgggggtaagggtatgactcagaccccacagcttcctggggccccgaAAAAACTGGTGGGGGTATCC	TNIP1	C		G	
rs5754217	^53^	Sequence forward: GGCAAGTCACTGGCTTCTTC Sequence reverse: CAAGGAAGCCAAATTGAGGA	UBE2L3	Insertion			
rs969985	^9^	Sequence forward: CAGCAGCCAGAGCTTACTGA Sequence reverse: ACAGCCAACACTGATTGCAC	BLK	Insertion

**Table 3:** List of rare SNPs that were identified.

Common SNP ID	Location GRCh37	RegulomeDB score	Associated Gene	Rare variant location	RegulomeDB score
rs9888739	chr16:31313253	4	*ITGAM*	chr16:31310306	4
rs10488631	chr7:128594183	3a	*IRF5*	chr7:128594188	3a
rs3823536	chr7:128579666	2b	*IRF5*	chr7:128579567	2b
rs12574073	chr11:128319478	3a	*ETS1*	chr11:128319490	2b
rs10036748	chr5:150458146	3a	*TNIP1*	chr5:150458365	2b
rs5754217	chr22:21939675-21939675		*UBE2L3*	chr22:21921209-21921364	
rs969985	chr8:11341870-11341870		*BLK*	chr8:11341211-11341453	

### Sanger sequencing

Sanger Sequencing was undertaken using Big Dye Terminator Chemistry version 3.1 (BDTv3.1) on a 3130xL capillary sequencer supplied by Applied Biosystems. The PCR products were purified using the Exo SAP protocol, in which 5 μL of PCR product is combined with 2 μL of EXOSAPIT enzyme mix from GE Healthcare. The reaction consisted of 1 cycle of 37 °C for 15 minutes, the enzyme was then heat inactivated for 15 minutes at 80 °C. The purified PCR products were then amplified in a sequencing reaction using the BDTv3.1 chemistry. The reaction mix consisted of 4.0 μL of 2.5x ready reaction mix, 2.0 μL of 5x Big Dye Sequencing Buffer, 1.0 μL of Forward or Reverse CRP primer at 3.2 pmol/μL, 2 μL of purified PCR product and 11 μL of DH2O. The cycling conditions were as follows: 1 cycle of 96 °C for 1 minute, 25 cycles of 96 °C for 10 seconds, 50 °C for 5 seconds, 60 °C for 4 minutes. Sequencing products were purified using the Ethanol/EDTA/Sodium Acetate Precipitation protocol in which 2 μL of 125 mM EDTA, 2 μL of 3 M sodium acetate and 50 μL of 100% ethanol was added to each sequencing reaction. The reaction was incubated at room temperature for 15 minutes and samples centrifuged for 2000–3000 g for 30 minutes. The supernatant was removed and 70 μL of 70% ethanol added and centrifuged at 1650 g for 15 minutes at 4 °C. The supernatant was removed and the samples were re-suspended in injection buffer and loaded on the 3130 capillary sequencer. The sequencing data was analysed using the software SeqScanner available from Applied Biosystems.

### Cell culture

Human B-lymphoblastoid cells (Raji cell line) were cultured in T75 flasks and grown overnight in 10 mL RPMI-1640 medium supplemented with 10% heat inactivated fetal bovine serum (FBS) in a tissue culture incubator humidified with a 5% CO_2_ at 37 °C.

### Generation of reporter contructs

The non-coding DNA variant fragments corresponding to the identified SNPs (Table 2) were constructed into the plasmid pGL4.27 [luc2P/minP/Hygro] (#E845A, Promega, Madison, WI).

### Luciferase assays

Plasmids constructed to bear the identified variants (3 µg/each) were transfected into 1 million Raji cells. After 24 h of transfection, cells were lysed and luciferase activities, as indicated by relative luminescence units (RLU) were determined using the luciferase assay system (#E1501, Promega, Madison, WI) according to the manufacturer’s instructions.

### ELISAs

ELISAs for MIF, IL-10 and IL-37 were performed as previously described^23,24,26^.

### Flow cytometry

Flow cytometric cell subset analysis in PBMC of healthy controls and patients with SLE is described elsewhere^22^.

## Supplementary information

Supplementary Table 1

## Data Availability

The datasets generated and/or analysed during the current study are available from the corresponding author on reasonable request.
