# Supplementary material for: Rare variants in non-coding regulatory regions of the genome that affect gene expression in systemic lupus erythematosus
Source: Sci Rep. 2019 Oct 28;9:15433. doi: 10.1038/s41598-019-51864-9 (PMC6817816; doi:10.1038/s41598-019-51864-9)
Supplement: Supplementary file 1 — Supplementary Table 1 [file 41598_2019_51864_MOESM1_ESM.pdf]

Supplementary Information

Rare variants in non-coding regulatory regions of the genome that affect gene expression in systemic lupus erythematosus

Sarah A. Jones[1#^], Stuart Cantsilieris[2#], Huapeng Fan[1], Qiang Cheng[1], Brendan E. Russ[3], Elena J. Tucker[4] James Harris[1], Ina Rudloff[5], Marcel Nold[5], Melissa Northcott[1], Wendy Dankers[1], Andrew E. J. Toh[1], Stefan J. White[2*], Eric F Morand[1*]

[1] Centre for Inflammatory Diseases, Department of Medicine, School of Clinical Sciences, Monash University, Clayton Victoria 3168, Australia

[2] Department of Molecular and Translational Science, Monash University, Clayton Victoria 3168, Australia

[3] Department of Microbiology, Biomedical Discovery Institute, Monash University, Clayton Victoria 3800, Australia

[4] Murdoch Children's Research Institute, Royal Children's Hospital, Parkville Victoria 3052, Australia; Department of Paediatrics, University of Melbourne, Parkville Victoria 3052, Australia

[5] Hudson Institute of Medical Research, Clayton Victoria 3168, Australia

[#,*]These authors contributed equally.

[^]Corresponding author: sarah.a.jones@monash.edu

**Expt repeat**	NT	pGL4.27 (vector only)	**IRF5 variant near rs3853236**			**ETS2 variant near rs12574073**			**TNIP1 variant near rs10036748**			**IRF5 variants near rs10488631**						
			WT	Variant	*P* value	WT	Variant	*P* value	WT	Variant	*P* value	WT (GG)	Variant (GA)	*P* value	Variant (AG)	*P* value	Variant (AA)	*P* value
	57	1388	11424	10424		8931	3077		14350	11963		35598	23476		25357		30819	
1	55	1303	10142	9750	*0.4*	8645	2733	*2E-05*	14171	10938	*0.0039*	34913	23430	*0.00024*	25351	*0.00036*	31190	*0.02*
	57	1393	9644	9371		8177	2861		13608	10522		32993	21916		24396		28476	
	16	256	1235	1736		677	374		4819	3938		7626	2185		2417		4596	
2	22	214	1106	1536	*0.0025*	635	350	*3E-05*	4601	3702	*0.017*	7537	2212	*0.00001*	2433	*0.00001*	4679	*0.0001*
	15	201	1149	1619		666	361		4280	3381		7041	2113		2204		4698	
	18	98	456	756		437	318		521	376		1596	1129		696		696	
3	17	73	404	626	*0.0074*	402	311	*0.0005*	542	391	*0.0011*	1946	1114	*0.0031*	771	*0.0006*	828	*0.001*
	14	87	365	620		419	312		493	357		1897	1033		735		832	
	27	83	367	649		418	276		492	343		1802	1098		783		812	
4	19	82	422	691	*0.0003*	433	344	*0.0052*	438	325	*0.0072*	2315	1060	*0.01*	752	*0.0034*	807	*0.0036*
	9	87	365	713		432	321		481	383		1724	1112		825		817	

Supplementary Table 1. Replicate luciferase assay results for cloned rare variants. Luciferase luminescence values obtained in repeated experiments in Raji cells 24 hours after transfection. NT – no transfection. P values obtained by Student's *t* test comparing rare variant alleles with the major wildtype (WT) alleles.
